# Supplementary material for: Genome-scale metabolic reconstructions of multiple Salmonella strains reveal serovar-specific metabolic traits
Source: Nat Commun. 2018 Sep 14;9:3771. doi: 10.1038/s41467-018-06112-5 (PMC6138749; doi:10.1038/s41467-018-06112-5)
Supplement: Supplementary file 1 — Supplementary Information [file 41467_2018_6112_MOESM1_ESM.pdf]

Genome-scale metabolic reconstructions of multiple *Salmonella* strains reveal serovar-specific metabolic traits

Seif et. al

Supplementary Notes:

Supplementary Note 1: Catabolic capabilities across closely related strains:

Closely related strains did not share similar catabolic capabilities indicating that gene loss came as a result of host adaptation. We observed that all Typhi and Paratyphi A strains lacked the capability to utilize galactonate as a sole carbon source and while Typhi strains are missing *dgoA*, *dgoK* and *dgoT* while Paratyphi A strains only lack *dgoK*. Zhou et. al argues that gene loss has occurred early in the history of Paratyphi A [1]. Additionally, Typhi and Paratyphi A strains are phylogenetically close and are known to be adapted to the same host. However the differential loss of genes involved in the same metabolic pathway suggests that independent convergence has occurred. We hypothesize that the loss of *dgoA* and *dgoT* may have occurred after serovar diversification and could have been driven by the lack of a need for galactonate transport in the human extra-intestinal environment.

Supplementary Note 2: Biological context for the loss of the catabolic capability for D-Tagatose:

D-tagatose is a natural sweetener that has been shown to be incompletely absorbed in the small intestine and subsequently fermented in the colon in both human, murine and other non-human primates [2]. It is also a product of human gut microbiota derived degradation of the mucus layer [3]. The tagatose transport and utilization operon consists of 9 ORFs. The disruption of 3 genes in the tagatose operon has been shown to be important for intestinal infection of cattle, chicken and pig but not intravenous infection of mice [4]. It was shown that knocking out one of those three genes results in abrogated growth of a *Salmonella* strain on M9 minimal medium + 1% D-tagatose [5]. Taken together these findings suggest that the microenvironment of extra-intestinal pathogens does not contain D-tagatose and that these pathogens are unaffected by the loss of the capability to uptake and utilize this nutrient.

Supplementary Note 3: Analysis of the growth predicted profiles that did not agree with experimental evidence

The first GEM for serovar Dublin strain CT_02021853 falsely simulated growth on proline and tricarballylate. The second GEM for serovar Agona strain SL483 falsely simulated an incapability to grow on glycolate and D-Tagatose and the third GEM for serovar Schwarzengrund strain CVM19633 falsely simulated growth on serine. We found that the false no growth phenotypes were due to the absence of homologous

sequences for the PTS tagatose transporters (STM325-STM3256) as well as a D-tagatose 1-phosphate kinase (STM3254) and glycolate oxidase (STM1620). Interestingly, an STM3254 deletion mutant was shown to be incapable of growth on M9 minimal medium + 1% tagatose suggesting that strain SL483 may have an alternative pathway for the utilization of tagatose [5]. Conflicting evidence was found for the successful utilization of glycolate across *Salmonella* strain. Thus far, there have been no molecular studies detailing out the glycolate utilization biochemical pathway in *Salmonella*. We notice that the annotated pathway in the *Salmonella* GEM mirrors the pathway that has been characterized in *E. coli* which means that it was probably added as a result of observed sequence homology of the genes involved in the pathway. Glycolate uptake and utilization is mediated by a multi-protein complex in *E-coli* (including *glcDEF*). However only one gene (STM1620) was found to correspond to such a function in *Salmonella*. Additionally, while it is annotated in the model as a glycolate oxidase, its genome annotation was that of a putative lactate oxidase. We noticed that STM1620 is only found in 46% of the genomes included in this data-set. We also found conflicting reports of the capability of several *Salmonella* strains (including str. LT2) to successful utilize glycolate [6–8]. Intriguingly, it was shown that STM1620 highly contributes to fitness of *Salmonella* strains across different hosts [9]. It is probable that the exact function of STM1620 was not correctly annotated in pan-STM.1.v2 , therefore we decided to assign to it a confidence score of 1 (as per the established protocol for building genome-scale reconstructions [10]). We also modified Figure 4.b (now 4.A) by excluding glycolate utilization as a differentiating metabolic capability because it could mislead the reader. The validation of predictions with experimental data precisely serves the function of highlighting such knowledge gaps.

Supplementary Note 4: A total of 531 nutrient environments highlight 242 conditionally essential genes:

To link genes in the reconstructed *Salmonella* pan reactome to the conferred catabolic capabilities, we searched for gene essentiality in all simulated nutrient environments. Here, we defined conditionally essential genes (CEG) as those genes found to be essential in one of the nutrient conditions but not in aerobic glucose+M9 minimal medium. Of the 531 nutrient environments 242 were anaerobic and 289 were aerobic. We identified a total of 242 predicted CEGs of which 195 and 217 were essential in at least one aerobic and at least one anaerobic nutrient condition respectively (with some genes being essential in both). Conditionally essential genes comprise genes involved in the uptake and catabolism of a nutrient source but also genes that participate in central metabolism. For instance, the four genes of the fumarate reductase complex (*frdABCD*) were conditionally essential in 82.2% of the anaerobic nutrient conditions

and several genes involved in glycolysis (including *tpiA*, *gapA*, *pgk, eno* and *ppc*) were classified as CEGs in various aerobic and/or anaerobic nutrient conditions (Fig 6A). Fumarate reductase is the terminal enzyme for anaerobic respiration and several catabolic pathways connect to central metabolism by feeding into glycolysis. Interestingly, phosphoenolpyruvate carboxylase (PPC) was essential in 85.1% of the simulated anaerobic conditions. PPC synthesizes oxaloacetate (a four carbon sugar) by adding bicarbonate to phosphoenolpyruvate (a three carbon sugar). It was shown that PPC Typhimurium mutants fail to grow on pyruvate or its precursors and seems to serve the only anaplerotic function by replenishing the supply of oxaloacetate which is drained away by the biosynthesis of amino acids from the tricarboxylic acid cycle [11]. Triose phosphate isomerase (TPI) was predicted to be conditionally essential in 30.4% of the simulated aerobic and anaerobic growth environments. Interestingly, TPI Typhimurium mutants were found to have decreased fitness in a mouse model of typhoid fever [12]. We further identified 78 and 72 CEGs that were essential in only one aerobic and anaerobic nutrient condition respectively. Xylose isomerase (*xylA*) is one such example that is categorized as CEG when the main carbon source is xylose. We refer to CEGs that are found to be essential in less than 5 nutrient conditions as sCEGs and those to be essential in more than 5 nutrient conditions as mCEGs. While sCEGs participate in nutrient catabolic pathways, mCEGs are located deeper in the network.

Supplementary Note 5: Heap's law and the comparison of pan genome curves: We constructed pan genome curves for four data-sets described in methods 1.D. We subsequently fit each of the curves with Heap's law using the "curve_fit" function from the Scipy toolkit[13].

$$f(N) = k\,N^{\gamma} + \varnothing$$

where N is the number of gene families, $\varnothing$ Is the average number of gene families per genome, and *k*, γ are the parameters to be fit. If γ is smaller than 1, the pan genome is said to be "closed" (meaning that after a sufficient number of genomes, no new additional gene families will be encountered). Based on fitting Heap's curve to the *Salmonella* pan genome curve (built from all 410 genomic sequences), we obtained a γ of 0.512 (**Fig. S3**). Previous efforts in the field concluded that *Salmonella* has a "closed" pan genome. However, when we built a pan genome curve from a subset of 41 genomes, we obtained a γ of 0.627. In order to identify the sensitivity of the Heap's law parameters, we fitted Heap's law to pan genome curves built using 10, 20, 30 and up to 410 genomes. The γ factor obtained by fitting Heaps' law to the sampled curves had a large standard deviation and decreased with the number of genomic sequences

included in the data set (**Fig. S8**) making the method of fitting Heap's law to the pan genome curve an unreliable predictor of the pan genome expansion (or the expected number of novel gene families encountered at a future genomic addition).

Supplementary Figures:

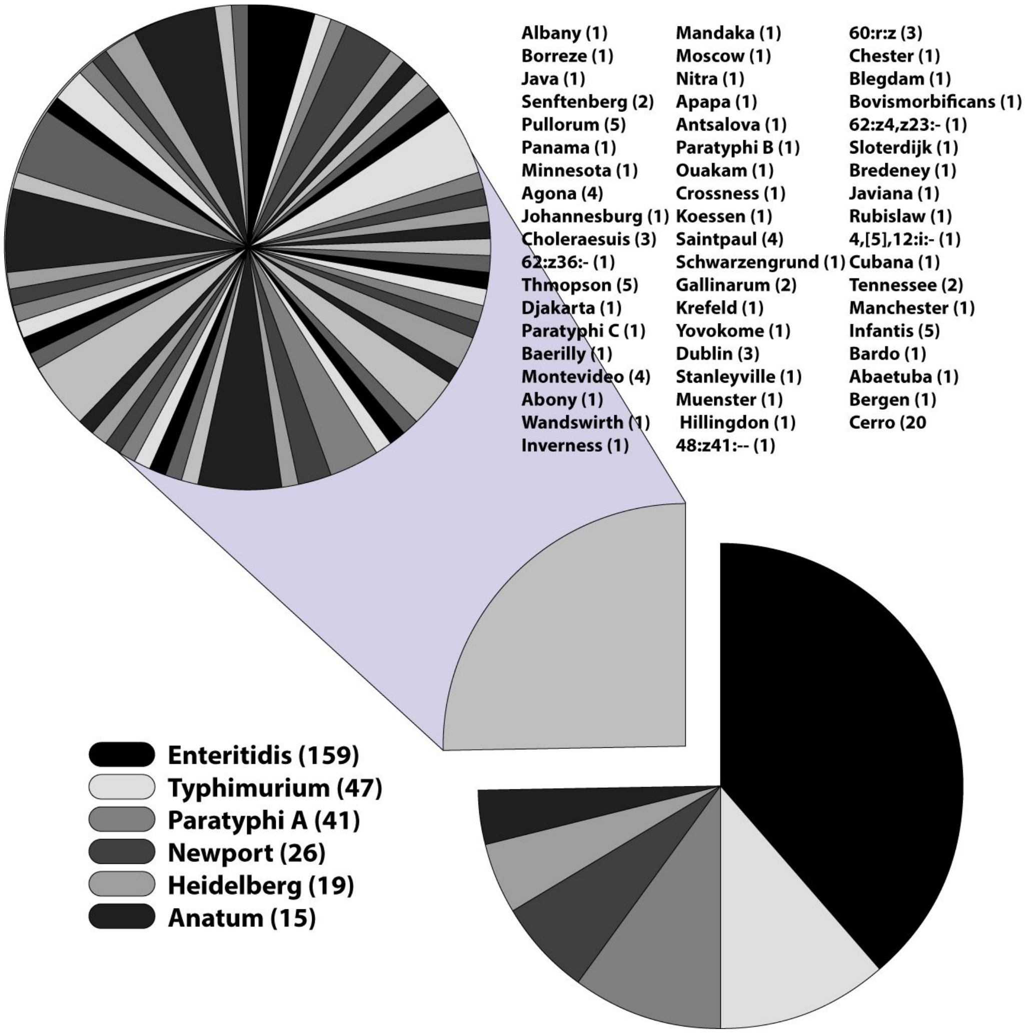

Supplementary Figure 1: Distribution of serotypes across the 410 selected genomic sequences: The data is highly skewed towards six serotypes: *S. Enteritidis, S. Typhimurium, S. Paratyphi A*, *S. Newport*, *S. Heidelberg* and *S. Anatum*

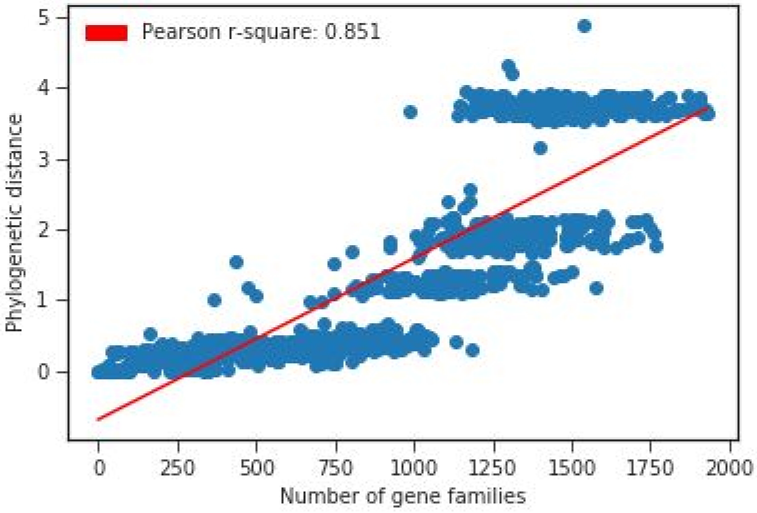

Supplementary Figure 2: Correlation between the phylogenetic distance and the size of the accessory genome of pairs of genomic sequences. The phylogenetic distance was computed from the alignment of the concatenation of the 7 housekeeping genes of *Salmonella* (aroC, dnaN, hemD, hisD, purE, sucA and thrA). The accessory genome of a pair of genome contains the gene families that are not shared between the two strains. We calculated the Pearson correlation between the two measurements of distance using the python command scipy.stats.pearsonr [14].

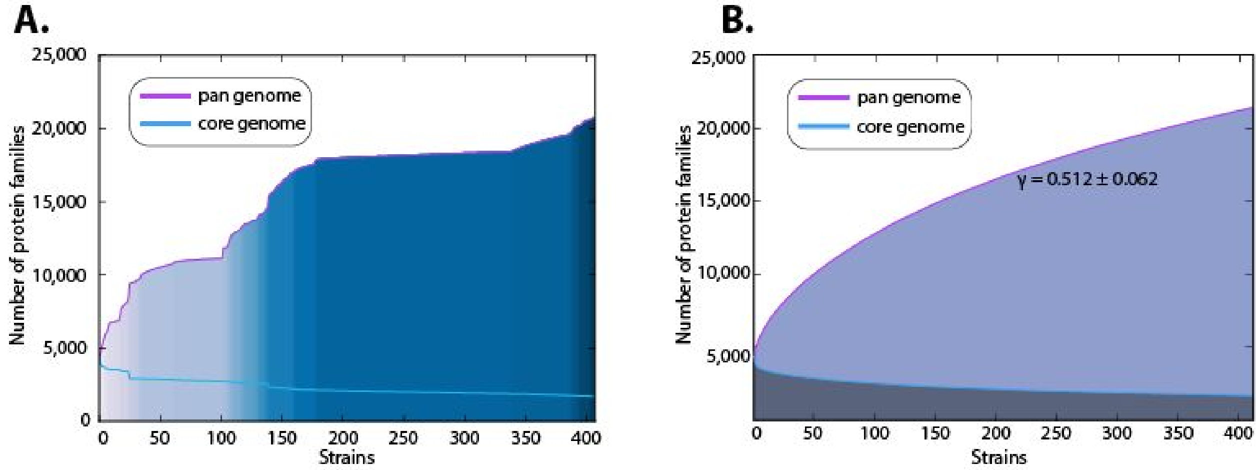

Supplementary Figure 3: Salmonella full pan genome: A) The *Salmonella* pan and core genome curves are plotted after the strains of a serovar are clustered together. Random sampling of the clustered strains is performed to obtain an average core and pan value after each genomic addition. This is the full version of Fig 1.A. Each shade of blue under the pan genome curve represents a new serovar. In other words, all strains of a serovar are represented by the same shade of blue. **B)** The *Salmonella* average pan and core genome curves are plotted after randomly sampling all 410 genomic sequences a thousand times. Heap's law was fitted to all of the 1000 sampled pan genome curve and the average and standard deviation of gamma is reported. This is a more traditional representation of pan and core genome curves and comes as an extension to the subplot in Fig 1.B for the *Salmonella* pan and core genome curves. The core genome is represented by a dark grey color and the accessory genome is represented in light mauve. The number of protein families shared across all genomes is plotted against the number of genomes added, i.e. the core genome curve (in light blue). The union of the set of gene families at each addition is plotted against the number of genomes added, i.e. the pan genome curve (in purple).

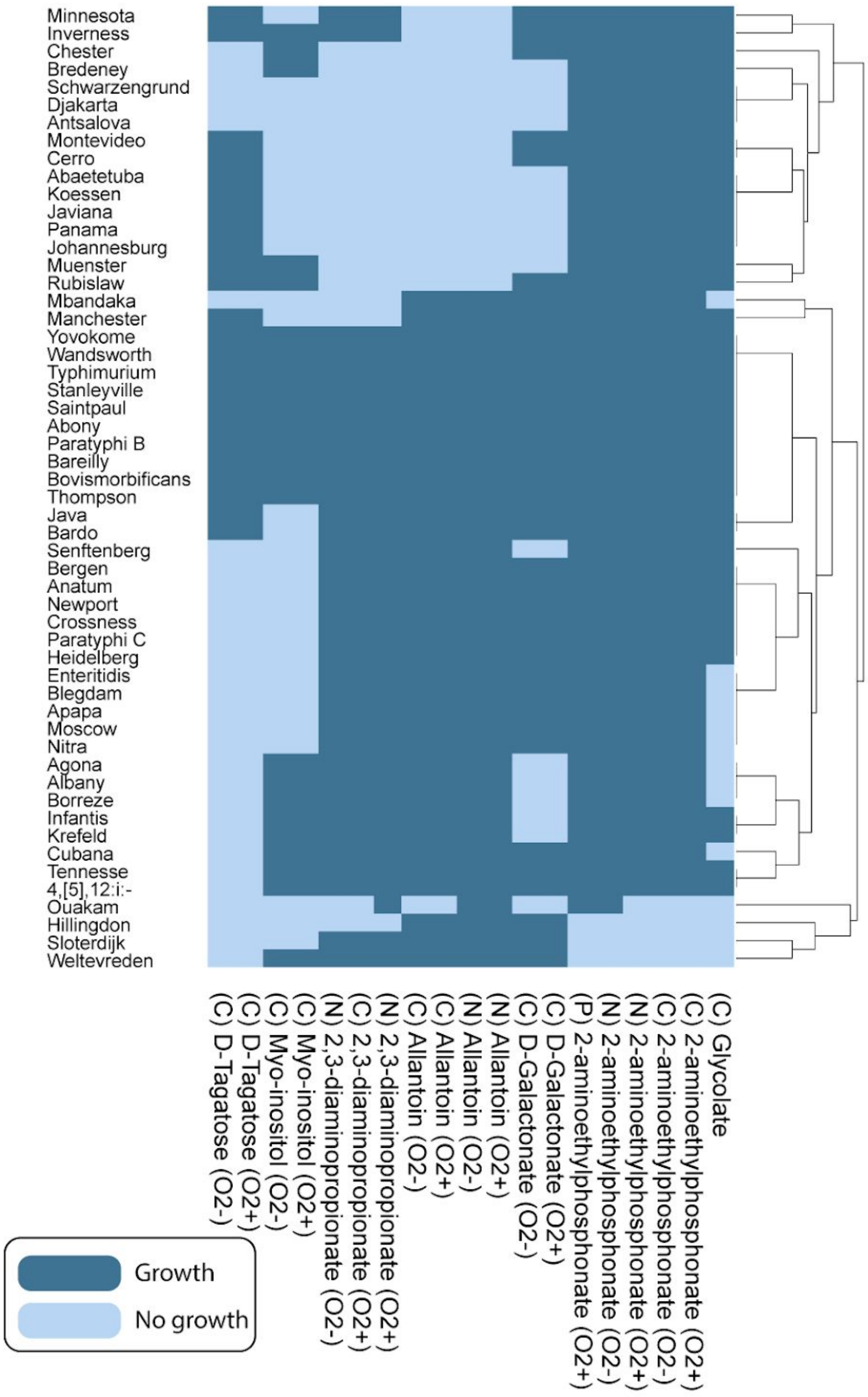

Supplementary Figure 4: Catabolic capabilities for 54 serovars of *Salmonella* listed across 22 nutrient conditions. A dark turquoise box represents predicted growth for a serovar in a nutrient environment and a light blue box represents the opposite. The nutrient conditions are listed with the nutrient source represented in parenthesis: C = carbon, P = phosphate, N = nitrogen. The catabolic capabilities were

tested in both aerobic and anaerobic conditions represented by O2+ and O2- respectively.

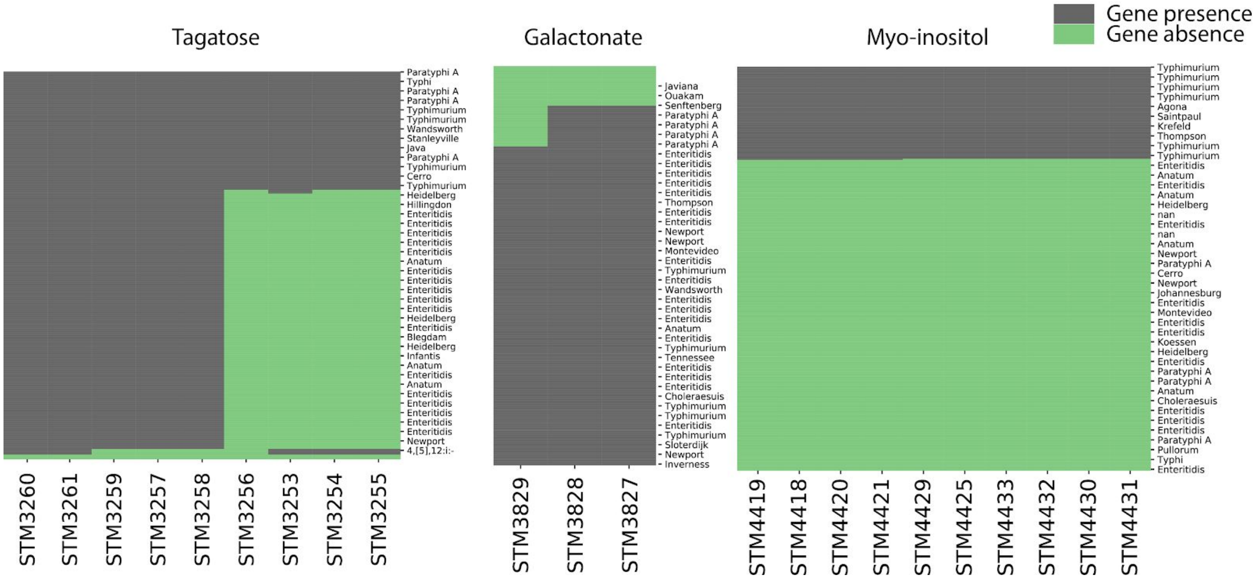

Supplementary Figure 5: Occurrence of genes of the myo-inositol, tagatose and galactonate utilization operons across 410 *Salmonella* strains.

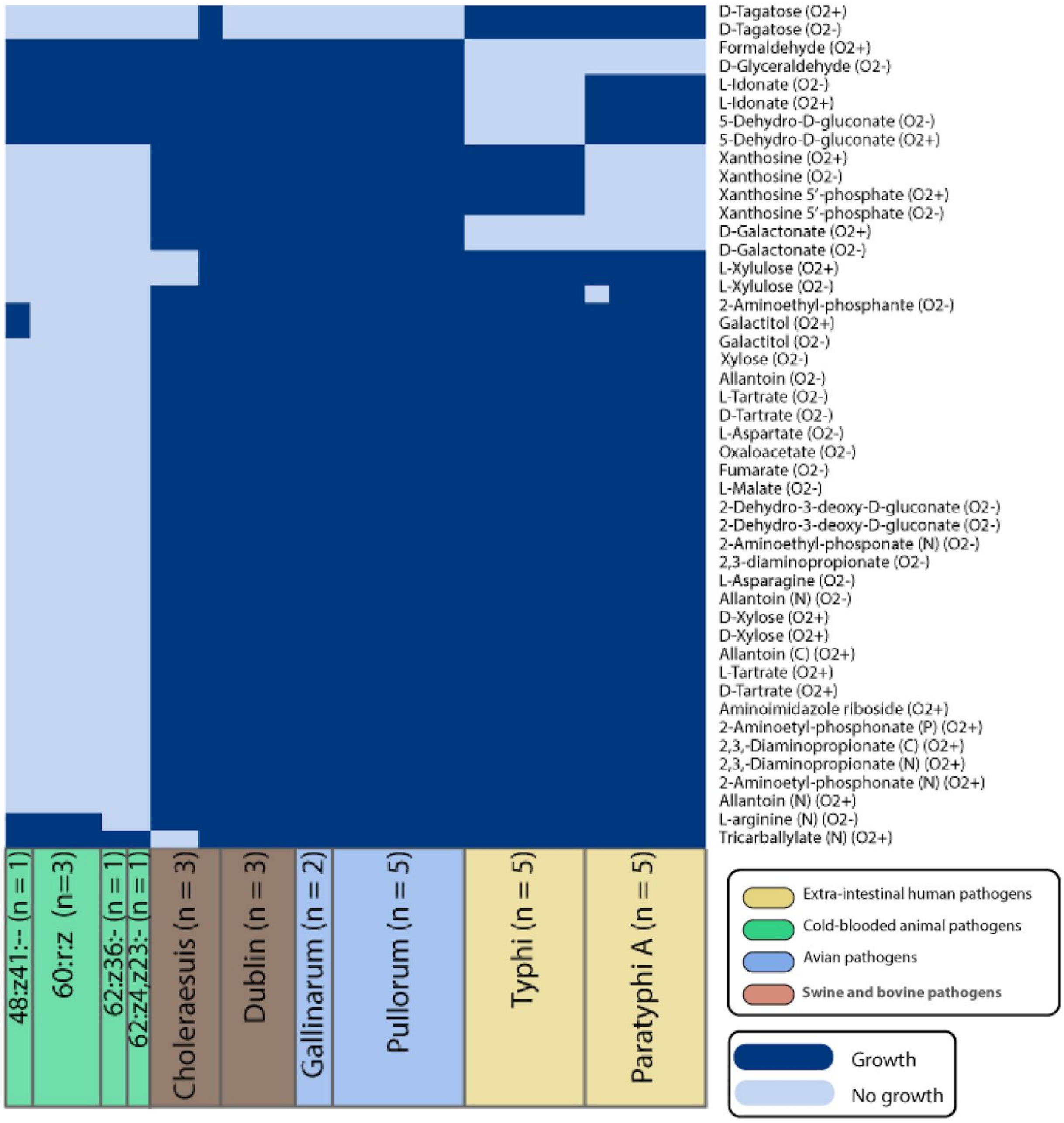

Supplementary Figure 6: Catabolic capabilities across host-specialists. Catabolic capabilities cluster host specialists together. A dark blue color indicates successful growth of a strain in a certain nutrient environment. The number of GEMs per serovar is listed in between parentheses. Colors indicate the host to which each serovar is specific.

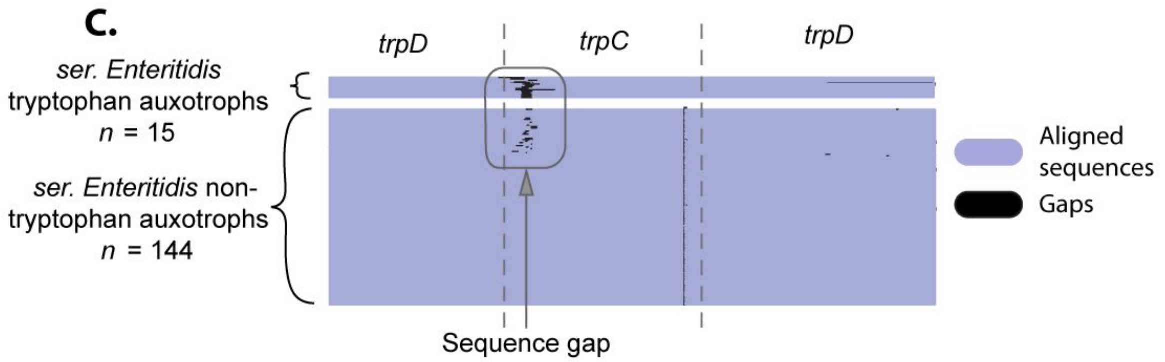

Supplementary Figure 7: Multiple sequence alignment of the *trp* operon across Enteritidis genomes. Gaps appear in black, aligned nucleotide sequences appear in blue. Note that upon closer examination of the multiple sequence alignment of the trp operon across several sequences, we noticed that a the some nucleotide bases in trpC were annotated as "N" which is the conventional letter used to signify that for each base pair position in this read, the sequencer was not able to determine if the base was A, T, C or G. As a consequence, the genome annotation platform did not detect an open reading frame in the region of trpC which was missing in the strain's genome scale reconstruction. This resulted in the strains being predicted to be auxotrophic. This is yet another way to use genome-scale reconstructions to refine genome annotations.

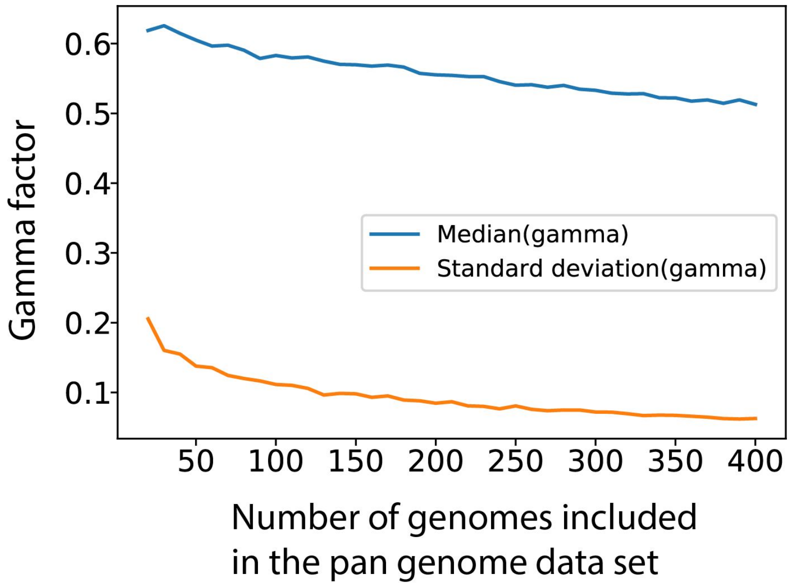

Supplementary Figure 8: Median and standard deviation of Heap's law derived gamma parameter as a function of the size of the data-set.

Supplementary Table 1: Characteristics of serovar-specific pan-genomes for 41 sampled genomes

Serovar (# genomes)	Average number of gene families per genome	Full pan genome size
S. Paratyphi A (41)	4331	4670
S. Typhimurium (47)	4661	7603
S. Enteritidis (159)	4337	4750
Salmonella (410)	4440	9473

Supplementary Table 2: Core and Pan metabolic reactome composition

Subsystem	Total Number of Reactions	Number of Core Reactions	% Conserved	% of Core Reactome	Number of Pan Reactions	% Pan Conserved
Other	144	89	0.618055556	0.046548117	55	0.127020785
Cell Wall/Membrane/Envelope Metabolism	246	182	0.739837398	0.095188285	64	0.147806005
Carbohydrate Metabolism	240	178	0.741666667	0.093096234	62	0.143187067
Inorganic Ion Transport and Metabolism	144	107	0.743055556	0.055962343	37	0.085450346
Cofactor and Prosthetic Group Metabolism	185	144	0.778378378	0.075313808	41	0.094688222
Inner Membrane Transport	325	253	0.778461538	0.132322176	72	0.166281755
Amino Acid	171	146	0.8538	0.07635983	25	0.05773

**Metabolism**			**0117**	**3**		**6721**
**Nucleotide Metabolism**	**173**	**150**	**0.8670 52023**	**0.07845188 3**	**23**	**0.05311 7783**
**Energy Production and Conversion**	**119**	**105**	**0.8823 52941**	**0.05491631 8**	**14**	**0.03233 2564**
**Lipid Metabolism**	**273**	**243**	**0.8901 0989**	**0.12709205**	**30**	**0.06928 4065**
**Outer Membrane Transport**	**325**	**315**	**0.9692 30769**	**0.16474895 4**	**10**	**0.02309 4688**
**Total**	**2345**	**1912**	**8.8620 01886**	**1**	**433**	**1**

Supplementary references:

1. Zhou, Z. *et al.* Transient Darwinian selection in Salmonella enterica serovar Paratyphi A during 450 years of global spread of enteric fever. *Proc. Natl. Acad. Sci. U. S. A.* **111,** 12199–12204 (2014).
2. Jayamuthunagai, J., Gautam, P., Srisowmeya, G. & Chakravarthy, M. Biocatalytic production of D-tagatose: A potential rare sugar with versatile applications. *Crit. Rev. Food Sci. Nutr.* **57,** 3430–3437 (2017).
3. Ravcheev, D. A. & Thiele, I. Comparative Genomic Analysis of the Human Gut Microbiome Reveals a Broad Distribution of Metabolic Pathways for the Degradation of Host-Synthetized Mucin Glycans and Utilization of Mucin-Derived Monosaccharides. *Front. Genet.* **8,** 111 (2017).
4. Chaudhuri, R. R. *et al.* Comprehensive identification of Salmonella enterica serovar typhimurium genes required for infection of BALB/c mice. *PLoS Pathog.* **5,** e1000529 (2009).

5. Nolle, N., Felsl, A., Heermann, R. & Fuchs, T. M. Genetic Characterization of the Galactitol Utilization Pathway of Salmonella enterica Serovar Typhimurium. *J. Bacteriol.* **199,** (2017).

6. Quan, J. A. *et al.* Regulation of carbon utilization by sulfur availability in Escherichia coli and Salmonella typhimurium. *Microbiology* **148,** 123–131 (2002).

7. Langridge, G. C. *et al.* Patterns of genome evolution that have accompanied host adaptation in Salmonella. *Proc. Natl. Acad. Sci. U. S. A.* **112,** 863–868 (2015).

8. Fricke, W. F. *et al.* Comparative genomics of 28 Salmonella enterica isolates: evidence for CRISPR-mediated adaptive sublineage evolution. *J. Bacteriol.* **193,** 3556–3568 (2011).

9. Chaudhuri, R. R. *et al.* Comprehensive assignment of roles for Salmonella typhimurium genes in intestinal colonization of food-producing animals. *PLoS Genet.* **9,** e1003456 (2013).

10. Thiele, I. & Palsson, B. Ø. A protocol for generating a high-quality genome-scale metabolic reconstruction. *Nat. Protoc.* **5,** 93–121 (2010).

11. Maeba, P. & Sanwal, B. D. Feedback inhibition of phosphoenolpyruvate carboxylase of Salmonella. *Biochem. Biophys. Res. Commun.* **21,** 503–508 (1965).

12. Paterson, G. K., Cone, D. B., Northen, H., Peters, S. E. & Maskell, D. J. Deletion of the gene encoding the glycolytic enzyme triosephosphate isomerase (tpi) alters morphology of Salmonella enterica serovar Typhimurium and decreases fitness in mice. *FEMS Microbiol. Lett.* **294,** 45–51 (2009).

13. Jones, E., Oliphant, T., Peterson, P. & Others. SciPy: Open source scientific tools for Python, 2014. *URL: http://www. scipy. org* **4,** (2014).

14. SciPy.org — SciPy.org. Available at: https://www.scipy.org/. (Accessed: 25th July 2017)
