# Supplementary material for: Genome-scale metabolic reconstructions of multiple Salmonella strains reveal serovar-specific metabolic traits
Source: Nat Commun. 2018 Sep 14;9:3771. doi: 10.1038/s41467-018-06112-5 (PMC6138749; doi:10.1038/s41467-018-06112-5)
Supplement: Supplementary file 3 — Description of Additional Supplementary Files [file 41467_2018_6112_MOESM3_ESM.docx]

- **Description of Additional Supplementary Files**
- File name: Supplementary Data 1
- Description: Genome accessions, strain and strain-specific reconstruction metadata including model and strain name, classification under specialist/generalist, number of metabolic reactions, metabolic genes and metabolites. The excluded sequences do not have strain-specific metadata.
- File name: Supplementary Data 2
- Description: Pan genome calculations; a) Calculated number of pan and core gene families per addition of a genomic sequence for 410 genomic sequences of Salmonella. b) Computed core and pan genome values for 1000 random samples of 41 strains of each of the 4 subsets used for the generation of figure 1B. Refer to methods section 1.C for a detailed explanation of the content of the subsets. c) Serotype-specific core gene families of Paratyphi A, Enteritidis and Typhimurium
- File name: Supplementary Data 3
- Description: Pan-reactome reconstruction; a) Novel reaction additions to the Salmonella metabolome. The reactions were extracted from BiGG based on sequence homology and manually curated against the literature for their presence in Salmonella. b) Functional annotation of Swissprot proteins with BiGG gene reaction rules. c) Pan-STM.v1.1 reactions. Contains all the metabolic reactions and processes included across all metabolic reconstructions of Salmonella. d) Pan-STM.v1.1 metabolites. Contains all the metabolites included across all metabolic reconstructions of Salmonella
- File name: Supplementary Data 4
- Description: Strain-specific model-driven nutrient catabolic predictions: a) Bidirectional best blast hit matrix; this matrix was used to determine presence/absence of a gene in a genome. b) Summary of the distribution of metabolic reactions and processes across 10 metabolic subsystems.c) In silico M9 minimal media formulation and carbon, nitrogen, phosphorous and sulfur nutrient sources used to simulate for different growth supporting environments. d) Predicted catabolic capabilities simulations across 410 GEMs across 290 growth supporting environment (aerobic). Growth is assumed for values above 0.01. e) Predicted catabolic capabilities simulations across 410 GEMs across 242 growth supporting environment (anaerobic). Growth is assumed for values above 0.01. f) List of the accession numbers for the strains whose catabolic capabilities are shown in figure 3B. 2
- File name: Supplementary Data 5
- Description: GEM validations: a) Experimental validation of growth profiles for 6 GEMS of Typhi across 86 nutrient sources, b) Experimental validation of growth profiles for 6 GEMs spanning 6 serovars of Salmonella across 59 nutrient sources
- File name: Supplementary Data 6
- Description: GEM-predicted strain specific auxotrophies/alternative pathway. A strain was predicted to be auxotrophic for a certain nutrient when it could not simulate growth in glucose + M9 minimal media alone.
- File name: Supplementary Data 7
- Description: Conditional gene essentiality: a) Conditionally essential genes (CEGs) in 532 nutrient conditions including 242 anaerobic and 282 aerobic conditions. b) Conditionally essential genes that were found to significantly contribute to fitness in at least one host and the number of times the genes are seen to be missing across strains of a serovar
